# Hemilingual edema as a warning sign for internal carotid artery dissection—a case report and literature review

**DOI:** 10.3389/fmed.2026.1765232

**Published:** 2026-03-09

**Authors:** Ioana Butnariu, Florentina-Melania Cojocaru, Vlad-Iulian Lăptoiu, Adriana Bidea, Iulia-Ana Maria Mitrică, Sorin Tuță, Dana Antonescu-Ghelmez, Florian Antonescu

**Affiliations:** 1 Carol Davila University of Medicine and Pharmacy, Bucharest, Romania; 2 National Institute of Neurology and Neurovascular Diseases, Bucharest, Romania; 3 Medikali Medical Center, Bucharest, Romania

**Keywords:** atypical stroke presentation, carotid dissection, hemilingual edema, tongue edema, tongue swelling

## Abstract

**Background:**

Internal carotid artery dissection (ICAD) is a rare but important cause of ischemic stroke in young adults. Its clinical presentation is highly variable, and atypical symptoms can delay recognition. Unilateral tongue (hemilingual) edema is an exceptionally uncommon manifestation. This case highlights its diagnostic relevance and explores potential pathophysiological mechanisms.

**Case presentation:**

A 23-year-old male presented with a four-day history of left-sided tongue swelling, occipital headache, and pulsatile tinnitus. Examination revealed left hemilingual edema and mild dysarthria, without motor or sensory deficits. Diagnostic workup included non-contrast cerebral CT, Doppler ultrasonography, MRI with contrast, CT angiography, and additional ENT, ophthalmological, laboratory, and cardiac evaluations. MRI demonstrated a subintimal hematoma of the left internal carotid artery extending from the distal C1 segment to the carotid canal, producing up to 80% stenosis, along with a small subacute ischemic lesion in the left centrum semiovale. The patient received antiplatelet therapy, resulting in symptomatic improvement within days. Follow-up MRI at 3 months confirmed complete arterial healing and minimal residual tongue swelling. The clinical picture suggested mild hypoglossal nerve involvement with sympathetic dysfunction but no motor impairment.

**Conclusion:**

Asymmetric tongue swelling may serve as an early and underrecognized sign of ICAD, particularly when accompanied by ipsilateral headache or cranial neuropathy. Early identification and appropriate imaging are essential to ensure timely diagnosis and reduce the risk of ischemic complications. Hemilingual edema likely results from sympathetic dysfunction due to local neurovascular involvement.

## Introduction

Carotid artery dissection (CAD) involves a longitudinal separation of the carotid artery wall layers, creating a false lumen. It is a potentially devastating condition that can lead to significant neurological morbidity. Typical causes include trauma and connective tissue disorders, but it is known to develop spontaneously (1). It usually occurs in the cervical region of the carotid artery (CA), a few centimeters above the bifurcation, from where it can extend to the intracranial segment (2).

Internal carotid artery dissection (ICAD) is often associated with ischemic events which can stem from thromboembolic complications or reduced blood flow. Presentation varies, but symptoms often include ipsilateral cervical or ocular pain, hemicrania (or a combination of the three), cervical bruits, hemiparesis, amaurosis fugax, Horner syndrome, and/or variable neurological deficits correlated with the severity of the resulting cerebral ischemia (3).

Diagnosis is established through advanced imaging techniques such as magnetic resonance angiography (MRA), computed tomography angiography (CTA), or conventional angiography (3).

Hemilingual edema is a rare type of presentation and in the absence of other signs, the diagnosis can be initially missed or delayed, with potentially severe consequences.

## Case description

We describe the case of a 23-year-old male, a non-smoker without notable medical history, who presented with a four-day history of left hemilingual edema, occipital headache, and left-sided pulsatile tinnitus. The initial symptoms also included brief, nonspecific visual disturbances such as bilateral mild image blurring, which had resolved before admission. There was no history of recent trauma or illicit substance use.

**Figure 1:**
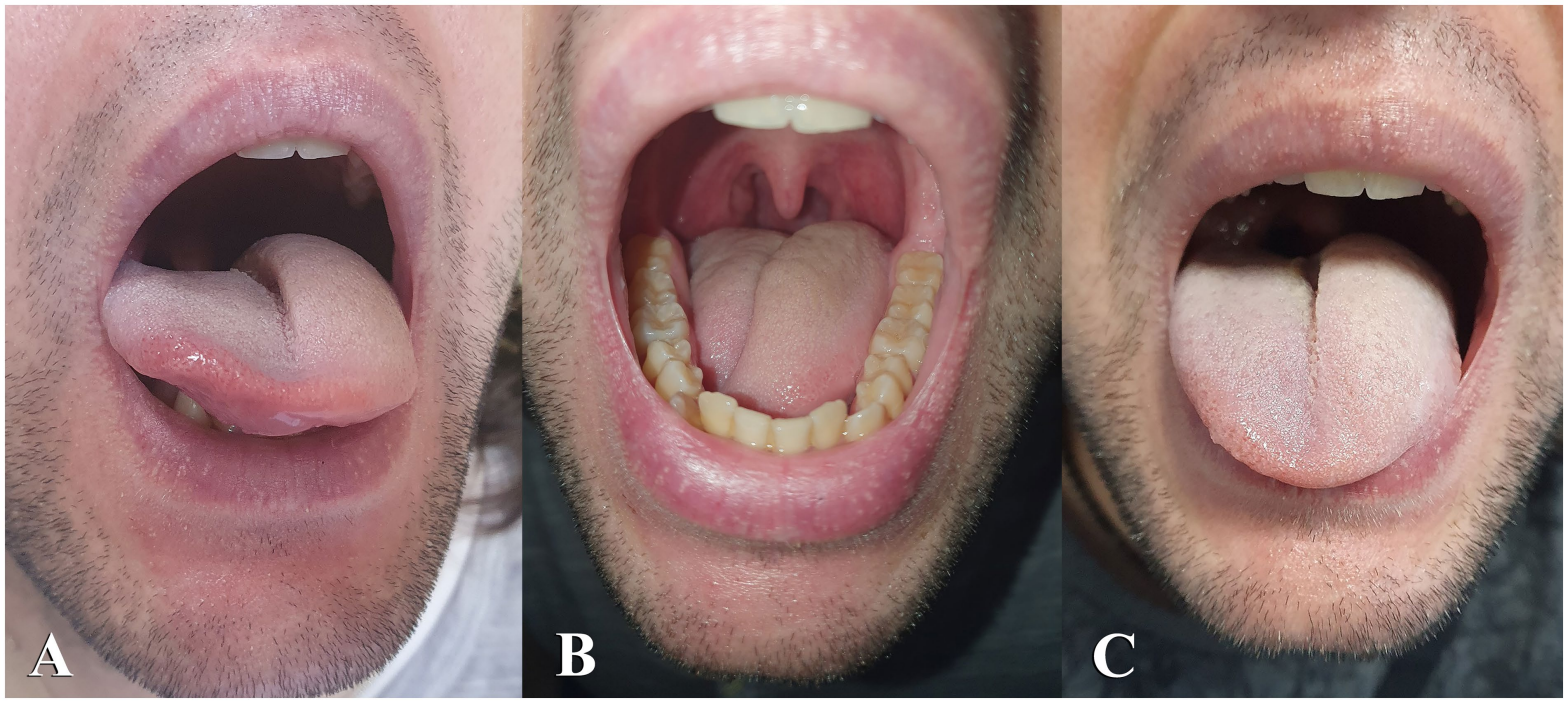
Swelling is present on the left side of the tongue upon admission. The tongue rests with deviation to the right **(B)** and maintains a midline position during protrusion **(A,C)**.

Physical examination revealed left hemilingual edema with the tongue deviating right at rest (Figure 1B). Despite this, tongue protrusion was midline (Figures 1A,C) and there was no evidence of tongue weakness. The patient exhibited minimal dysarthria, which he attributed to the swelling, but no other focal neurological deficits. Routine blood tests were unremarkable, so was thyroid function and a screening for systemic vasculitides.

**Figure 2:**
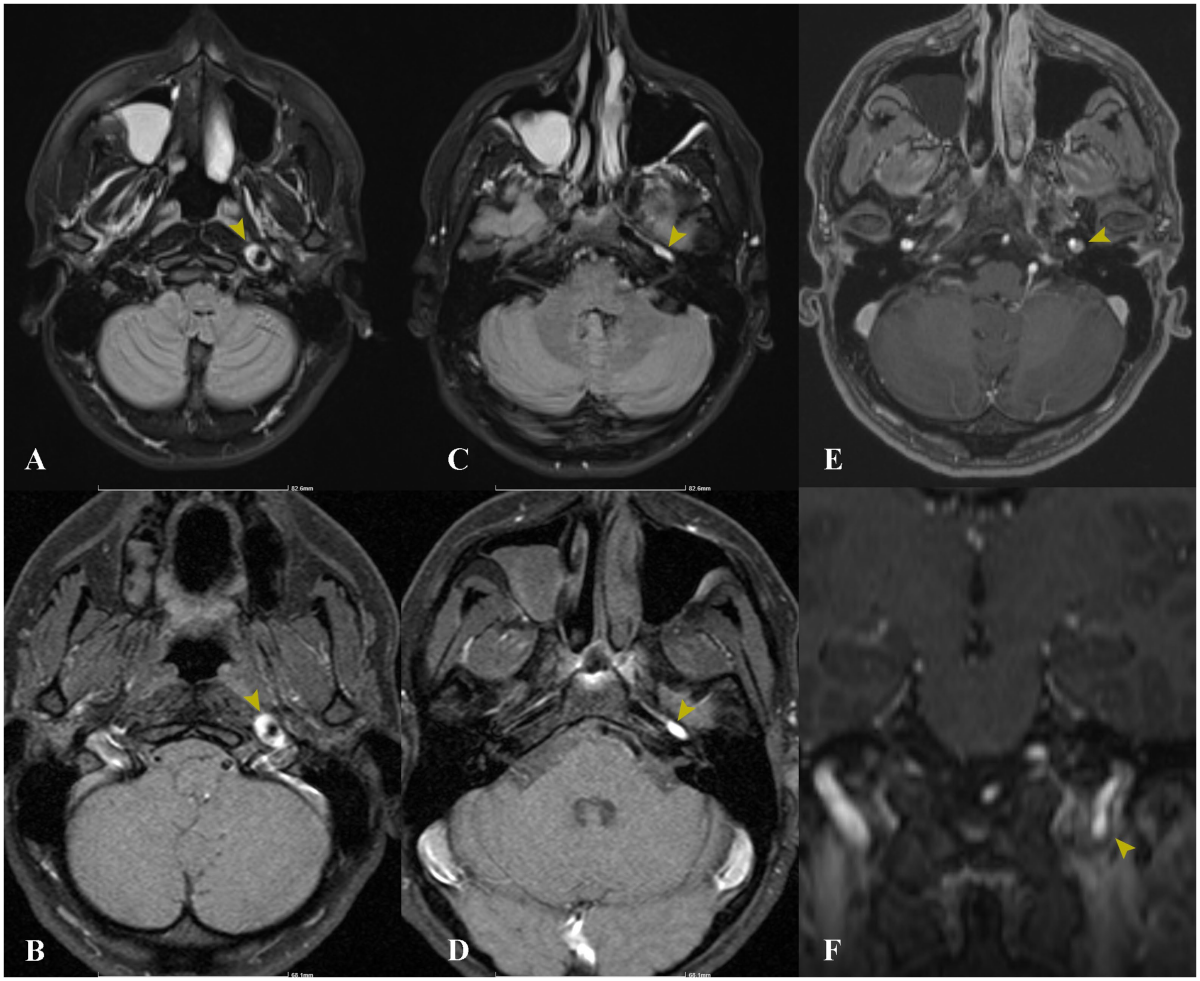
Axial MRI sequences, including FLAIR **(A,C)** and native T1 **(B,D)**, demonstrate a high signal intensity corresponding to the mural hematoma at the level of the left internal carotid artery (indicated by yellow arrows). Axial **(E)** and coronal **(F)** MRI T1 contrast-enhanced sequences reveal the dissection flap and residual lumen (yellow arrows).

**Figure 3:**
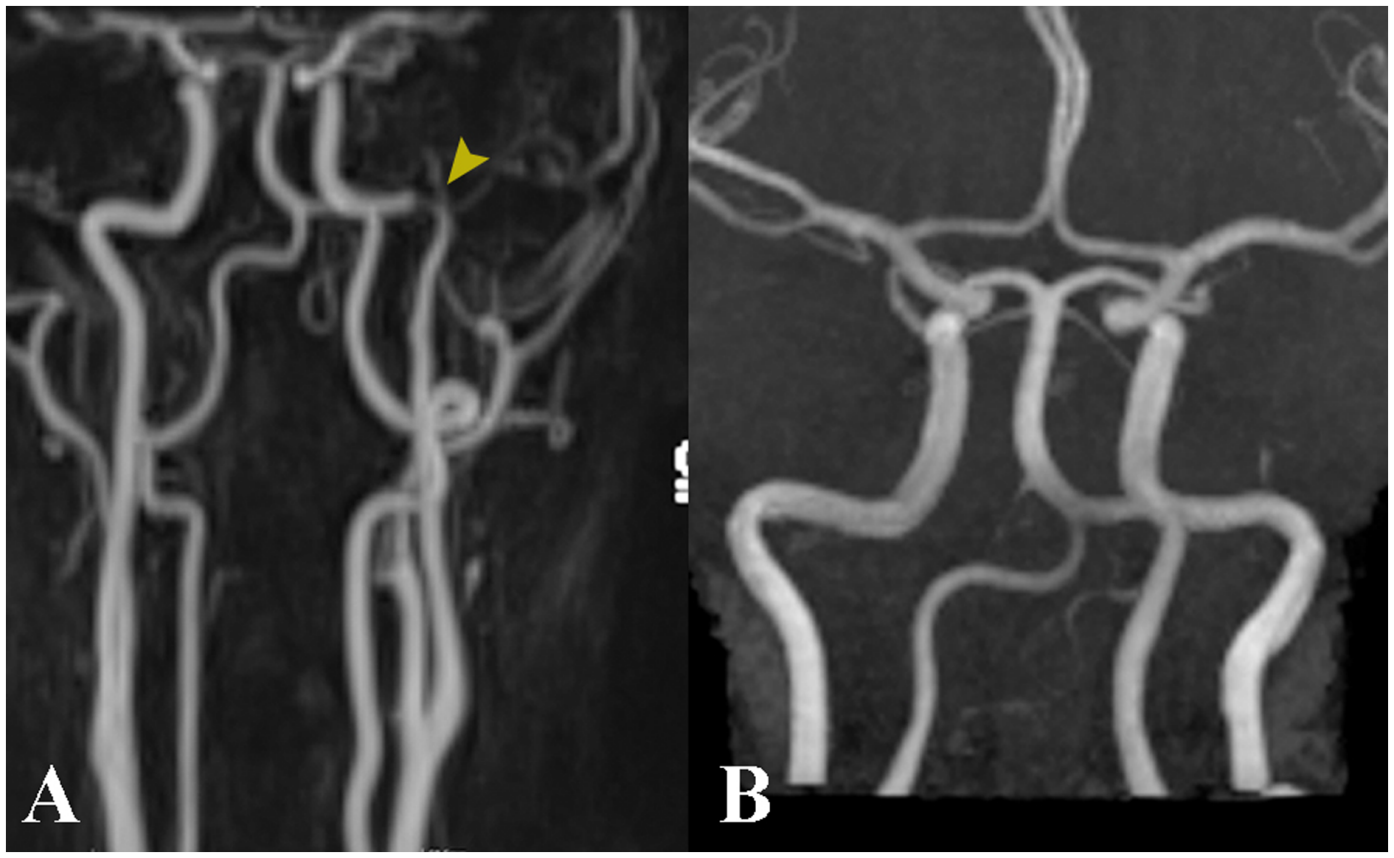
Contrast-enhanced angioMRI at admission **(A)** reveals a narrowing of the intrapetrous segment of the left internal carotid artery (yellow arrow). At the three-month follow-up, angiographic findings have returned to normal **(B)**.

**Figure 4:**
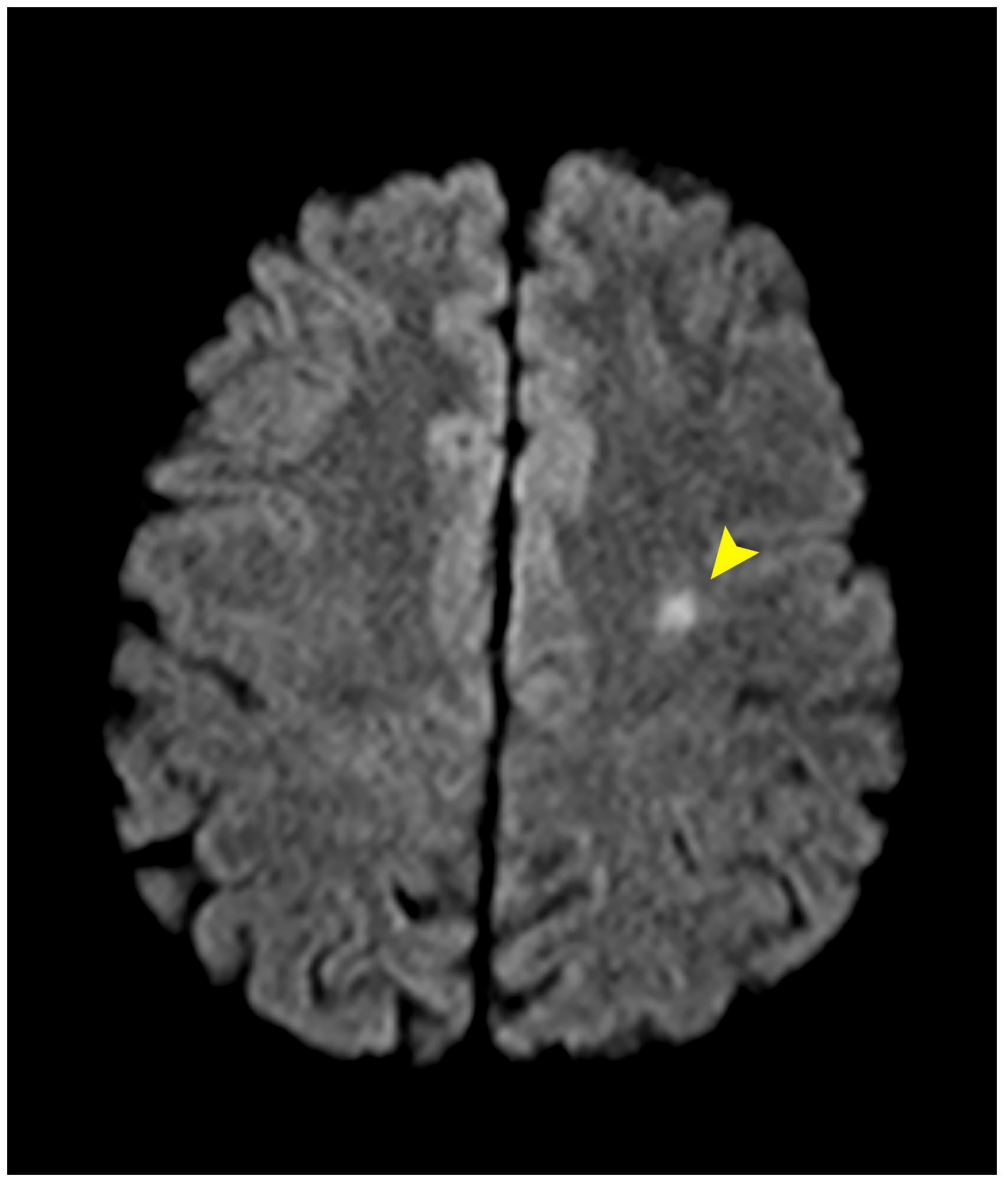
Axial MRI-DWI sequence demonstrates a small hyperintense lesion in the left centrum semiovale (arrow), exhibiting features consistent with acute ischemia.

Imaging with non-contrast cerebral CT and Doppler ultrasonography showed no significant abnormalities. Given the clinical suspicion for ICAD, a contrast-enhanced cerebral MRI was ordered (Figures 2, 3A). Imaging revealed a subintimal hematoma along the left ICA, extending from the distal C1 segment up to the carotid canal, spanning approximately 2 cm. This led to progressive luminal narrowing, with up to 80% stenosis noted at the level of the carotid canal (Figure 3A). Additionally, a small (7 × 5 mm), T2/FLAIR hyperintense lesion in the left centrum semiovale, with moderate diffusion restriction, was identified, consistent with a subacute ischemic event in the left middle cerebral artery territory (Figure 4).

To further characterize the ICAD, a CT angiography was ordered, which confirmed the lesion originating at the distal cervical C1 segment and extending into the proximal petrous C2 segment, with a maximal stenosis of 80%–85%.

Ophthalmological assessment, including a comprehensive fundus examination, revealed no abnormalities. Cardiac evaluation, including an electrocardiogram and transthoracic echocardiography reported normal findings. Additionally, an ENT consultation was obtained, which identified hypomobility of the left side of the tongue, due to the edema, and a deviated nasal septum as the only notable observations.

**Figure 5:**
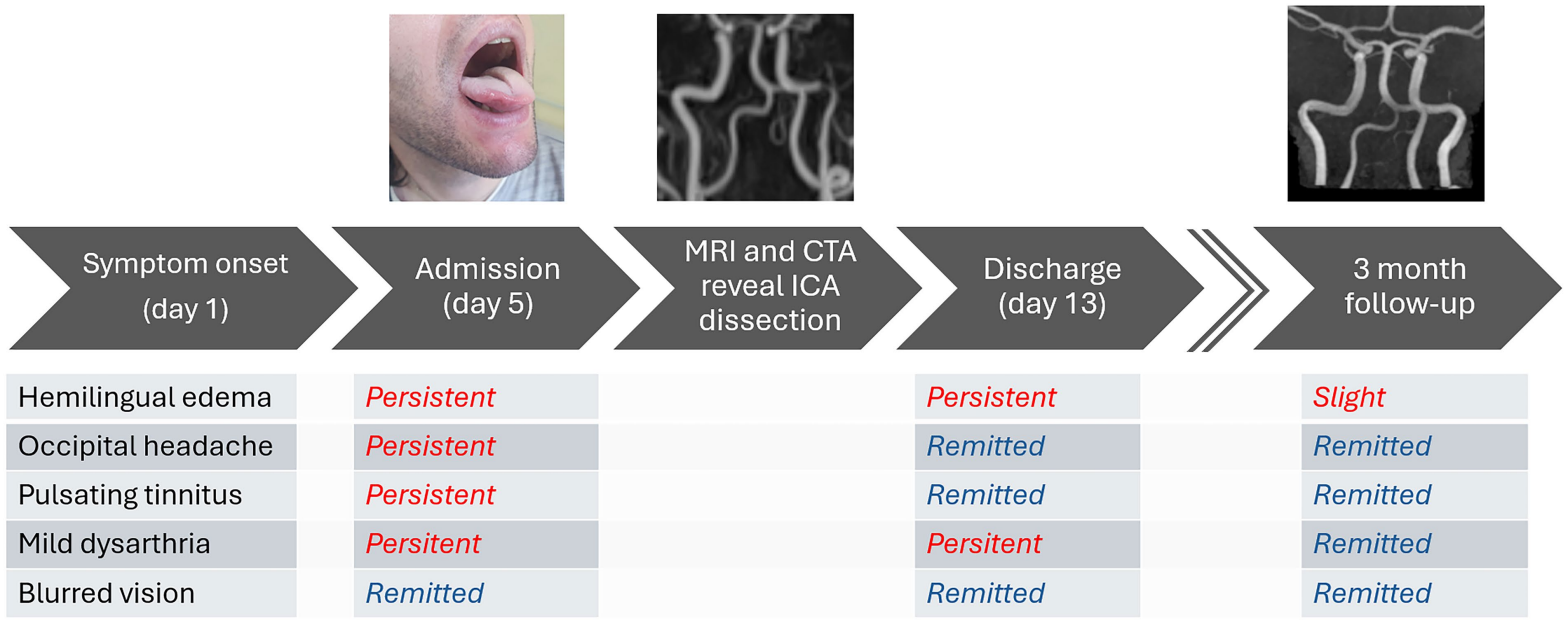
Timeline illustrating the progression of symptoms and MRI findings at diagnosis, as well as at the three-month follow-up.

The patient was administered antiplatelet therapy with aspirin for secondary prevention of ischemic events, in addition to analgesics. Both headache and tinnitus resolved within a few days, and the patient was subsequently discharged. At the three-month follow-up, only minimal residual left hemilingual edema was observed; the neurological examination was unremarkable, and the patient reported no further complaints. A follow-up MRI demonstrated restoration of the artery to normal condition (Figure 3B). We included a timeline figure to illustrate the patient’s clinical course throughout follow-up (Figure 5).

## Discussion

ICAD can have a large spectrum of clinical manifestations, extending from asymptomatic or nonspecific pain syndromes of the head and neck to massive cerebral stroke. Common symptoms include local pain, Horner syndrome, pulsatile tinnitus and cranial neuropathies, especially involving the lower cranial nerves. Hemilingual edema is an unusual and often under-recognized manifestation of ICAD. We are aware of only seven other reports in the current literature (1–10), which we have reviewed in Table 1.

**Table 1:** Summary of all published case reports describing patients presenting with lingual edema in the context of carotid artery dissection (CAD), detailing the site of dissection, demographic characteristics, associated symptoms (stroke, Horner syndrome, tinnitus, visual manifestations, pain), and clinical evolution.

Differential diagnosis	Arguments against in this case
Allergic angioedema (histamine-mediated)	No allergy history; no identifiable trigger; no response to antihistamines; prolonged duration
Non-allergic angioedema (bradykinin-mediated)	No ACE inhibitor exposure, no personal or family history, symptoms persisted longer than typical
Infectious/non-infectious glossitis	No local or systemic sign of infection; negative inflamatory markers
Cranial nerve pathology (XII nerve involvement)	No motor deficit
Sympathetic dysfunction secondary to carotid artery pathology	Most consistent with clinical course and vascular findings

Left-sided ICA dissections represented the majority (5 out of 7 patients), with a strong male predominance (6 out of 7 patients). All but one patient with a history of cocaine use, had no significant medical history to point in the direction of concomitant vascular pathology (11).

The anatomical distribution of the dissection in the reviewed cases consistently involved the extracranial segment of the internal carotid artery, though with some variability in the precise location along its course. Most cases (5 out of 7 patients) were explicitly described as extracranial, with one case detailing a dissection extending from the C2 vertebral level to the intrapetrous segment, and another localized to the C1–C2 segment (12, 13).

Interestingly, half of the cases, including the present one, initially sought medical attention shortly after symptom onset but were misdiagnosed with allergic reactions and prescribed corresponding medication, highlighting a potential diagnostic pitfall in early presentations (12, 14–16).

Unlike our patient, none of the reported cases had radiographic or clinical evidence of ischemic stroke, chronic or acute, suggesting variability in clinical severity. Furthermore, none of the patients reported tinnitus or visual disturbances, and only three described unilateral hemicrania at symptom onset (12, 13, 16).

Horner syndrome, a classic but inconsistently present feature of carotid dissection, was documented in only one case, which was also notable for concomitant lower cranial nerve involvement (13). Notably, none of the patients had a history of diabetes mellitus, consistent with the profile of spontaneous, non-atherosclerotic dissection. These observations underscore the heterogeneity in clinical presentation and the potential for initial misdiagnosis in spontaneous ICA dissection.

Presentation as hemilingual edema can be a confounding factor in acute stroke scenarios, as it may mimic an allergic reaction or other types of angioedema. This distinction is especially important because intravenous thrombolysis - particularly with alteplase - can precipitate a non-allergic, bradykinin-mediated angioedema which sometimes can also be asymmetrical (4).

Unilateral tongue edema presents a broad differential diagnosis, encompassing both allergic and non-allergic angioedema, infectious glossitis, other inflammatory processes, and cranial nerve pathology. In this instance, a comprehensive immunological assessment—including evaluation of complement levels and C1 esterase inhibitor—was not conducted. Nevertheless, several clinical findings were inconsistent with angioedema as the underlying cause. The patient reported no personal or family history of allergies or angioedema, had no exposure to angiotensin-converting enzyme inhibitors or other medications associated with bradykinin-mediated reactions, and demonstrated neither systemic nor local signs of infection, with negative inflammatory markers. Furthermore, the symptoms did not respond to antihistamine therapy administered prior to admission and persisted significantly longer than is typical for angioedema.

The underlying pathogenesis remains unclear, but the prevailing hypothesis involves sympathetic dysfunction leading to vasodilation and secondary edema. The precise anatomical point where this happens is not well established. Most sympathetic fibers reach the area via the perivascular sympathetic plexus along the external carotid and lingual arteries. Therefore, dissection of the common or external carotid artery could theoretically induce such a dysfunction, though attributing this to ICAD is more challenging. Our observations are summarized in Table 2.

**Table 2:** Primary differential diagnoses for hemilingual edema, along with relevant clinical, laboratory, and imaging considerations applicable to our patient.

Article	Site of dissection	Patient data	Horner syndrome	Tinnitus	Visual manifestations	Pain	Stroke	Follow-up
Siniscalchi et al. (11)	Left extracranial ICAD	M, 44 y/o	−	−	−	−	−	Complete regression of ICA dissection at 3 months with improvement of both tongue swelling and weakness
Kaushik et al. (12)	Left ICAD starting at C2 extending in the intrapetrous segment	M, 44 y/o	−	−	−	−	−	No follow-up
Lindsay et al. (20)	Left skull base ICAD	M, 44 y/o	−	−	−	−	−	Complete resolution of the dissection at 5 months with clinical resolution 2 months after
Ryan et al. (14)	Right extracranial ICAD	M, 44 y/o	−	−	−	−	−	No follow-up
Felix et al. (15)	Left ICAD, intrapetrous segment	M, 52 y/o	−	−	−	+	−	No follow-up
Pawlukowska et al. (13)	Left ICAD C1–C2 segment	F, 36 y/o	+	−	−	+		Partial regression of the intramural hematoma of the left ICA with no significat narrowing of the arterial lumen
Flux et al. (16)	Right ICAD	M, 48 y/o	−	−	−	+	−	Normal lumen revascularization at 5 months, headaches and hypoglossal paresis had decreased

Hypoglossal nerve palsy secondary to ICAD is a recognized phenomenon with proposed mechanisms including direct nerve compression from mural hematoma and nerve ischemia due to compromised blood supply (5, 6). There exists the possibility that postganglionic sympathetic fibers originating in the superior cervical ganglion travel within the hypoglossal nerve. While animal models support this concept, evidence in humans remains limited (7). Still, branches connecting the superior cervical ganglion and hypoglossal nerve have been described (17) (p. 469).

Tongue edema has rarely been reported in association with acute hypoglossal nerve lesions (approximately 1% of cases), typically occurring alongside tongue paralysis (8, 9). There are also reports of tongue pseudohypertrophy resulting from motor denervation in hypoglossal neuropathies, characterized by fatty replacement of denervated muscle (10). This is invariably a gradual process, which was not observed in our patient, nor were any MRI changes noted at admission or at the three-month follow-up.

While a temporal and anatomical association between hemilingual edema and internal carotid artery dissection was observed in our case, as in the previously mentioned reports, a direct causal relationship cannot be conclusively established. The proposed mechanism of sympathetic dysfunction—potentially mediated through involvement of the hypoglossal nerve or adjacent perivascular sympathetic fibers—remains hypothetical and is mainly supported by anatomical considerations and limited prior case reports rather. Of other possible mechanisms, we would like to point out that local venous congestion secondary to regional mass effect was considered less likely in the present case due to normal jugular vein appearance on the angiography. The lack of inflammatory markers or infectious signs made an inflamatory process unlikely.

In our patient’s case, we believe the edema was due to minor injury to the hypoglossal nerve that led to sympathetic dysfunction but did not cause motor deficits. The cause of the mild swelling that persists 3 months later, despite a normal MRI of the ICA, is still uncertain, a possible explanation being a lasting low-level sympathetic dysfunction.

The optimal antithrombotic strategy in internal carotid artery dissection remains a subject of ongoing debate. Both antiplatelet therapy and anticoagulation have been shown to be effective in preventing recurrent ischemic events, with randomized trials and meta-analyses demonstrating no clear superiority of one approach over the other (18).

At the time of this patient’s presentation in 2023, prevailing guidelines and available evidence supported the use of either antiplatelet agents or anticoagulation, with treatment individualized according to clinical severity, infarct burden, and bleeding risk (19). In the present case, the ischemic lesion was small, there was no evidence of recurrent embolization, and the patient exhibited rapid clinical improvement. For these reasons, antiplatelet therapy was selected as a safe and appropriate therapeutic option.

## Patient’s perspective

I’m 25 and hardly ever see doctors. First came a dull ache in the back of my head. Then the left side of my tongue puffed up, and I heard a whoosh in my left ear, like my heartbeat. At first the tests did not show much, which was scary. The MRI found a problem with a neck vessel and a tiny stroke. I was given aspirin and the medical team provided reassurance. The noise and pain faded in a few days. A three-month check-up indicated no further problems, and everything has been fine since.

## Conclusion

Asymmetric tongue swelling should prompt medical professionals to consider ICAD as a possible diagnosis, particularly when accompanied by cranial nerve deficits or ipsilateral headache. This condition may be confused with thrombolysis-associated angioedema; however, its presence prior to infusion, restriction to one side of the tongue, and frequent occurrence alongside hypoglossal nerve palsy can help differentiate between the two. Prompt diagnosis and treatment can prevent or limit life-threatening complications such as stroke. The mechanism of hemilingual oedema accompanying ICAD is still incompletely understood with theories mainly positing postganglionary sympathetic fiber involvement in the carotid plexus or, more probably, in the hypoglossal nerve.

## Data Availability

The original contributions presented in the study are included in the article/supplementary material, further inquiries can be directed to the corresponding author.
